# Characterization of Heterogeneity and Spatial Distribution of Phases in Complex Solid Dispersions by Thermal Analysis by Structural Characterization and X-ray Micro Computed Tomography

**DOI:** 10.1007/s11095-016-1923-3

**Published:** 2016-04-19

**Authors:** Muqdad Alhijjaj, Samy Yassin, Mike Reading, J. Axel Zeitler, Peter Belton, Sheng Qi

**Affiliations:** 1 School of Pharmacy, University of East Anglia, Norwich, Norfolk UK NR4 7TJ; 2 Department of Pharmaceutics, College of Pharmacy, University of Basrah, Basrah, Iraq; 3 Department of Chemical Engineering and Biotechnology, University of Cambridge, Pembroke Street, Cambridge, CB2 3RA UK; 4 Department of Chemical Sciences, University of Huddersfield, Queensgate, Huddersfield, UK HD1 3DH; 5 School of Chemistry, University of East Anglia, Norwich, Norfolk UK NR4 7TJ

**Keywords:** drug-polymer miscibility, hot melt extrusion, injection moulding, phase separation, solid dispersions, thermal analysis by structural characterization (TASC), XμCT tomography

## Abstract

**Purpose:**

This study investigated the effect of drug-excipient miscibility on the heterogeneity and spatial distribution of phase separation in pharmaceutical solid dispersions at a micron-scale using two novel and complementary characterization techniques, thermal analysis by structural characterization (TASC) and X-ray micro-computed tomography (XμCT) in conjunction with conventional characterization methods.

**Method:**

Complex dispersions containing felodipine, TPGS, PEG and PEO were prepared using hot melt extrusion-injection moulding. The phase separation behavior of the samples was characterized using TASC and XμCT in conjunction with conventional thermal, microscopic and spectroscopic techniques. The *in vitro* drug release study was performed to demonstrate the impact of phase separation on dissolution of the dispersions.

**Results:**

The conventional characterization results indicated the phase separating nature of the carrier materials in the patches and the presence of crystalline drug in the patches with the highest drug loading (30% *w/w*). TASC and XμCT where used to provide insight into the spatial configuration of the separate phases. TASC enabled assessment of the increased heterogeneity of the dispersions with increasing the drug loading. XμCT allowed the visualization of the accumulation of phase separated (crystalline) drug clusters at the interface of air pockets in the patches with highest drug loading which led to poor dissolution performance. Semi-quantitative assessment of the phase separated drug clusters in the patches were attempted using XμCT.

**Conclusion:**

TASC and XμCT can provide unique information regarding the phase separation behavior of solid dispersions which can be closely associated with important product quality indicators such as heterogeneity and microstructure.

**Electronic supplementary material:**

The online version of this article (doi:10.1007/s11095-016-1923-3) contains supplementary material, which is available to authorized users.

## Introduction

Solid dispersions have been used to improve the dissolution properties of poorly water-soluble drugs in an attempt to achieve better oral bioavailability and overall therapeutic outcomes (1–3). These dispersions have been often loosely classified into single-phase molecular dispersions and phase separated systems with varying degrees of structural complexity (4–6). Phase separation of and the formation of microstructures in the solid dispersions are the result of the diversity in the physicochemical properties of the drugs and excipients used in the formulations, which affects their miscibility. Traditionally phase separation has often been considered as an example of instability or incompatibility between the drug and excipients and therefore been avoided in industrial formulation development (7,8). This is largely a result of a lack of understanding regarding the mechanisms of the formation and ability to control of the progression of phase separation. More recently however, intentionally forming phase separated solid dispersions to improve stability or modulate the drug release profile has been proposed (9–11). From the literature, the most commonly observed phase separation behavior in solid dispersions is the separation of the incorporated drug from the carrier polymer and excipient materials (if more than one carrier material was used) (9,10) as either amorphous or crystalline domains (12–14).

Although conventional characterization techniques, such as differential scanning calorimetery (DSC and MTDSC), powder X-Ray diffraction (PXRD) and spectroscopic methods including IR, Raman and terahertz spectroscopy, often allow the confirmation of presence of phase separation, understanding the phase separation behavior in solid dispersions can still be challenging. The overlapping diffraction patterns or spectra from different phases or the thermal dissolution of one phase into another during heating in the DSC often lead to the difficulty in accurate data interpretation (15–17). Many excipients and active ingredients are organic materials which makes scanning electron microscopy (SEM) measurements in combination with element dispersive spectroscopy (EDS) powerless for identifying detailed phase separation due to the lack of elemental variability between samples. In addition, the conventional characterization methods mentioned above have not been able to effectively provide information on two important aspects of phase separation in formulations, heterogeneity and the 3D spatial distribution of different phases. Addressing these two aspects of phase separated solid dispersions will advance our understanding of how to control the formation and kinetics of phase separation behavior in complex solid formulations and in turn enable the rapid development of phase-separated dispersions which may be used for the delivery of multiple active pharmaceutical ingredients in one formulations. The motivation behind this study is to investigate these two less understood features of phase separation in solid dispersions by applying two novel characterization methods, thermal analysis by structural characterization (TASC) and X-ray micro computed tomography (XμCT), alongside conventional analytical tools.

This study introduces the use of two non-conventional methods, thermal analysis by structural characterization (TASC) and X-ray micro computed tomography (XμCT), that are not commonly used for studying pharmaceutical solid dispersions. We have evaluated the potential of these techniques for characterizing heterogeneity and spatial distribution of phase separations in solid dispersions. TASC is a thermal microscopic analysis method recently developed by Reading *et al.* with a particular focus on studying the glass transition kinetics and thermal dissolution behavior of materials (18). TASC is an optical analogue of micro/nano thermal analysis which has been reported in the literature for studying the phase separation behavior of solid dispersions (19). Micro/nano thermal analysis can pin-point the different phases present in the dispersion by identifying the differences in their thermal transition temperatures using heated AFM tips. The recent development of local nano-thermal analysis into an imaging method, transition temperature microscopy (TTM), has demonstrated the capacity of allowing the mapping of phase separation in some dispersion formulations (9,19). However, the disadvantage of micro/nano thermal analysis and TTM is that the measurements are often time consuming. Instead of using AFM as the measurement platform in micro/nano TA, TASC uses conventional, user-friendly hot stage microscopy with novel algorithm for quantifying changes in successive micrographs of the samples during heating or cooling. The detailed working principle of TASC has been explained previously (18,20). The subtle changes in the samples appearance in the course of heating or cooling detected by TASC can then be converted into thermal transition graphs. Alhijjaj and co-workers reported the first use of TASC for pharmaceutical applications and identified the advantages of TASC including rapid measurement and high sensitivity for detection of subtle thermal transitions and heterogeneity of the samples (20).

XμCT is a 3D X-ray imaging technique that has been widely used in a diverse range of disciplines to study the microstructure of objects without causing damage to the original sample. In contrast to X-ray diffraction methods, where X-rays are not absorbed but are reflected by an ordered array of matter, with a XμCT experiment it is the absorption of X-rays that results in the image, in a manner analogous to transmission microscopy. The differentiation of different phases by XμCT relies on the electron density differences that are characteristic of different elements. In the pharmaceutical industry, XμCT is used routinely to identify physical imperfections in solid dosage forms showing a high density contrast such as voids and cracks in tablets and coatings (21,22). Therefore the ability of the technique to distinguish materials with similar attenuation coefficients such as amorphous and crystalline forms of the same drug can be extremely limited (23) unless synchrotron radiation is used to improve the phase contrast (24,25). However for the conventional XμCT used in this study, in theory, if sufficient electron density differences are present between different phases contained within a sample, XμCT should be effective for resolving the distribution of these phases in 3D. The distribution of solid excipients in compressed tablets has been studied using XμCT based on this principle (26). However it has not been widely used to investigate phase separation in solid dispersions (27).

In this study a series of complex solid dispersions were prepared containing a poorly soluble model drug, felodipine, two semi-crystalline polymers, polyethylene glycol (PEG) 4000 and polyethylene oxide (PEO) 900,000, and semi-crystalline D-α tocopheryl polyethylene glycol 1000 succinate (Vitamin E TPGS). The dispersions were prepared by hot melt extrusion-injection moulding (HME-IM) to provide buccal patches containing felodipine, which would avoid its extensive first pass hepatic metabolism when administered orally, improving its bioavailability and allowing a reduced dose to be given via the buccal route (28). The rationale for the selection of excipients is that PEG allows the patches to be formed easily by HME, PEO provides mucoadhesive properties and TPGS acts as a drug permeation enhancer and solubilising agent (29–33). As a result of the limited miscibility between the excipients and felodipine as well as the semi-crystalline nature of the polymers used, the HME-IM patches showed phase separation. The presence of chlorine (Cl) in felodipine molecules provides an electron density difference between pure drug clusters and the rest of the excipients. When a significant amount of felodipine is dissolved in the excipients, the contribution of higher electron density of felodipine molecules allows identification of the drug-rich domains in the dispersions using XμCT. The technique cannot be used to distinguish between crystallised and amorphous drug only regions of high drug concentration however with complementary information provided by techniques such as PXRD and ATR-FTIR the crystalline/amorphous nature of phase separated drug domains can be confirmed. In addition, we report a preliminary attempt of using XμCT as a quantitative method to estimate the amount of drug phase separation in processed patches.

## Materials and Methods

### Materials

Felodipine was purchased from Afine Chemicals Ltd (Hangzhou, China). Polyethylene glycol (PEG) 4000 was purchased from Sigma Aldrich (Poole, UK). Polyethylene Oxide (PEO) WSR 1105 (MWT = 900,000) was kindly donated by Colorcon Ltd (Dartford, UK). Vitamin E TPGS was kindly donated by BASF (Ludwigshafen, Germany).

### Hot Melt Extrusion and Injection Moulding (HME-IM)

The extruder used in the fabrication of felodipine patches was a twin-screw bench-top hot melt extruder with a set of co-rotating conical screws (HAAK MiniLab II Micro Compounder, Thermo Electron, Karlsruhe, Germany). The extruder was connected to an injection moulding apparatus (HAAKE MiniJet System, Thermo Electron Corporation, Karlsruhe, Germany). Before processing, physical mixtures of the formulations were prepared at different drug loadings (see Table I). The physical mixtures were prepared by initially mixing crystalline felodipine in the molten TPGS (65°C) followed by the addition of the other excipients. The semi-solid mixtures were further blended thoroughly using a mortar and pestle for at least 2 min at room temperature. This mixture was then fed into the extruder, the barrel temperature of which was pre-set to 65°C and 100 rpm with 5 min of residence time. After extrusion, the extrudate was loaded into the pre-heated cylinder of the injection moulding apparatus (65°C) and injected into a patch shaped mould (25 mm × 25 mm × 0.5 mm), warmed to the same temperature as the cylinder using 300 bars as an injection pressure for 20 s. The patches were allowed to cool inside the mould for 1 h prior to collection.

**Table I:** Compositions of Felodipine HME-IM Solid Dispersion Patches

Loading (*w/w*)	Felodipine	PEG 4000	PEO WSR 1105	Vit E TPGS
Placebo	0%	40%	30%	30%
10%	10%	36%	27%	27%
20%	20%	32%	24%	24%
30%	30%	28%	21%	21%

### Scanning Electron Microscopy (SEM) and Energy-Dispersive X-ray Spectroscopy (EDS)

Surfaces and cross sections of the freshly prepared patches were scanned using the JSM 5900LV Field Emission Scanning Electron Microscope (Jeol Ltd, Japan) equipped with a tungsten hairpin electron gun and operating at an acceleration voltage of 5–20 kV. As the samples were relatively soft, dipping the samples into liquid nitrogen and crushing the frozen samples in order to obtain the natural morphology of the cross-sections of formulations with various drug loadings. Both kinds of sample were fixed on sample stubs using double adhesive tape. A Polaran SC7640 sputter gold coater (Quorum Technologies, Newhaven, UK) was used to coat the surfaces and cross-sections prior to imaging. EDS (INCA Energy manufactured by Oxford Instruments) connected to the SEM was used to map the distribution of drug clusters using Cl in felodipine as the marker. Samples were tested using both SEM and mapping mode EDS (data can be found in Supplementary Information).

### Attenuated Total Reflectance Fourier Transform Infrared (ATR-FTIR) Spectroscopy

A IFS 66/S FTIR spectrometer (Bruker Optics Ltd, Coventry, UK) fitted with a Golden Gate® ATR accessory with temperature controllable top plate (Specac Orpington, UK) and diamond internal reflection element was used to identify the physical form of felodipine and the possible interaction between the drug and the other excipients included in the patches. All samples were scanned using the following parameters: 2 cm^−1^ resolution, 32 scans for sample and background, and 4000–550 cm^−1^ spectrum range in absorption mode. The spectra of 3 replicates per sample for all drug loadings were analyzed using OPUS software.

### Powder X-Ray Diffraction (PXRD)

PXRD was used in this study to identify the polymorphic form of felodipine in the different formulations and the possible transformation from one physical form to another as the drug loading percentage increased from 10 to 30% *w/w*. In addition, the analysis was also used to investigate the effect of the drug on the crystallinity of PEG-PEO. All measurements were performed using the Thermo ARL Xtra X-ray diffractometer (Thermo Scientific, Switzerland) equipped with a copper X-ray Tube (*λ* =1.540562 Å). All PXRD patterns were obtained using an X-ray beam generated with an acceleration voltage of 45 kV and a current of 40 mA. The angular scan range was 5 ° < 2Ɵ < 60 ° with a step width of 0.01° and scan speed of 1 s/step.

### Differential Scanning Calorimetry (DSC)

Thermal analysis of felodipine loaded patches, their physical mixes and the raw materials was performed using the Q-2000 MTDSC (TA Instruments, Newcastle, USA) equipped with a RC90 cooling unit. Full calibration was performed prior to the samples measurements. For samples scanned using standard DSC, a heating rate of 10°C/ min and a heating range of −80–180°C were used. Before scanning, 2–3 mg of samples were weighed accurately and crimped in standard DSC pans (TA Instruments, Newcastle, USA). The obtained thermograms were analyzed using the Universal Analysis software. All measurements were performed in triplicate.

### Thermal Analysis by Structural Characterisation (TASC)

The TASC system was composed of a temperature controlled heating/cooling Linkam MDSG600 automated stage fixed to a Linkam imaging station that was attached to a microscope working in reflective mode (LED light source and × 10 magnification lens) and was equipped with a digital camera to capture images that correspond to thermal events as a function of temperature. For cooling ramps, the temperature of the stage is controlled using a cooling unit that operates by purging liquid nitrogen into the stage.

For all samples analyzed, thin slices of the prepared patches (0.6–1.2 mm × 0.6 mm × 0.2 mm) were cut using a sharp blade and placed in standard DSC pans (TA Instruments, Newcastle, USA). A pre-designed temperature program (10°C/min) for heating, cooling and reheating cycles with an isothermal period of 1 min separating the ramps was applied to the prepared samples. Before starting the experiments, the image-capturing mode was activated at an image acquisition rate of 1 frame/ °C. The captured images were then collected and analyzed using the TASC software provided by Cyversa (Norwich, UK). The results obtained were statistically analyzed by using one-way analysis of variance (ANOVA). Statistical significance was accepted at the *p* ≤ 0.05 level.

### X-Ray Micro-Computed Tomography (XμCT)

A SkyScan1172 high-resolution X-ray micro computed tomography (XμCT) scanner (Bruker-microCT, Kontich, Antwerp, Belgium) was used to analyse felodipine solid dispersions with different drug loading percentages (0–30% *w/w*). The analysed samples were imaged using an aluminium filter to cut-off high energy X-rays at an isotropic voxel resolution of 3 μm over a total of 20 min acquisition time and a subsequent image reconstruction time took approximately 20 min per sample, using the NRecon program (version 1.6.8.0, Bruker-microCT). The reconstructed images were analysed using CTan and CTvol software in which the images for a small section (designated as a region of interest ROI) for each sample are converted to binary images followed by thresholding each component according to differences in density and represented in 3D models. Powder compacts made of the physical mixtures of crystalline felodipine and the rest of the excipients with consistent compositions to those used in the HME-IM formulations were prepared for the quantitative studies. The compacts (13 mm in diameter) were prepared by compressing (500 mg) of the premixed physical blends into flat-faced disks using an IR press (Specac, Kent, UK) with 10 kN pressure held for 5 min.

### *In Vitro* Drug Release Studies

Unidirectional dissolution studies to simulate the release profile for systemic buccal administration were conducted using the paddle over disc method (similar to USP apparatus 5) using a dissolution apparatus (Caleva 8ST, Germany). Under non sink conditions, patch samples having the equivalent of 10 mg of felodipine (maximum daily dose) attached to a glass disc using double adhesive tape were immersed in 900 ml of phosphate buffer saline pH 6.8 (simulated salivary fluid) at 37 ± 0.5°C and 100 rpm paddle rotation. At different pre-determined time intervals, 5 ml samples were withdrawn and filtered using a 0.45 um filter unit (Minisart NML single use syringe, Sartorius, UK). The filtered samples were then diluted with an equal volume of absolute ethanol and the samples were analysed using a UV–VIS spectrophotometer (Perkin-Elmer lambda 35, USA) at 363 nm. Samples withdrawn were substituted with dissolution media at the same temperature after each sample was taken. The details of the dissolution methodology development and validation are described in Supplementary Information. All drug release studies were conducted in triplicate.

## Results and Discussion

### Conventional Microscopic, Spectroscopic and Thermal Characterization of Phase Separation in HME-IM Patches

**Fig. 1:**
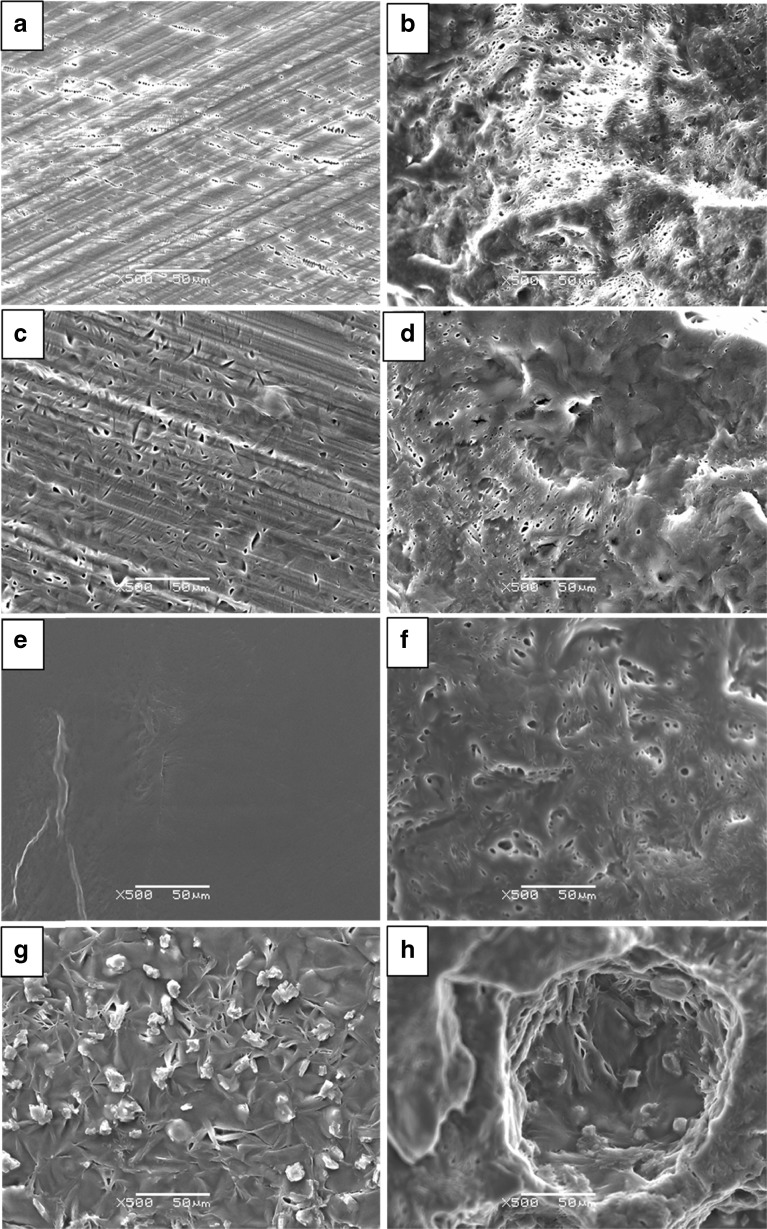
SEM images surfaces (**a**), (**c**), (**e**), (**g**) of the placebo patches, patches with 10%, 20% and 30% felodipine loading and their corresponding cross-sections (**b**), (**d**), (**f**), (**h**).

**Fig. 2:**
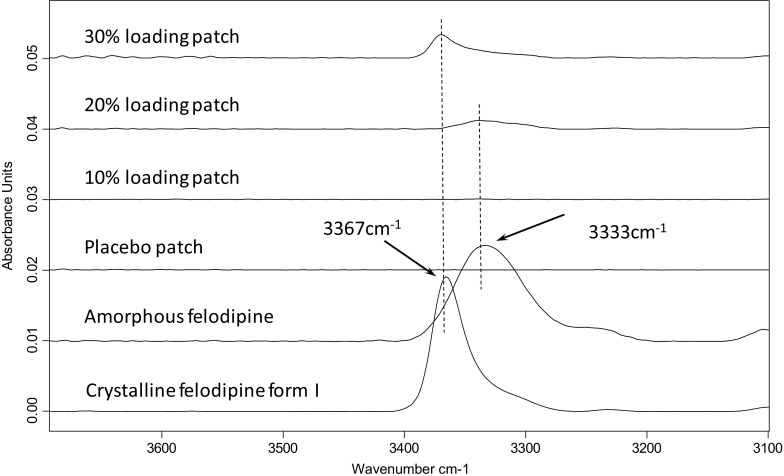
Partial ATR-FTIR spectra of felodipine NH stretching region of the HME-IM patches with different drug loadings in comparison to crystalline and amorphous felodipine.

Images captured using SEM (Fig. 1) revealed that the surfaces of the solid dispersion patches, except for those with 20% *w/w* drug loading, show the presence of small cracks and air voids and increased roughness with increasing the drug loading. The cross-sectional images of the patches show increased roughness in the interior in comparison to the surfaces and a clear porous character for all samples. Large air pockets between 100 and 300 μm in diameter, and particles (often with defined edges), with diameters of 10–20 μm, can be observed only in the patches with 30% drug loading. EDS analysis using chlorine (Cl) as the marker for felodipine (Supplementary Information Figure S1) confirmed that these particles contain a higher concentration of felodipine than other areas. With the confirmation of the presence of crystalline felodipine by PXRD (see Supplementary Information Figure S2), these high felodipine concentration areas are likely to be crystalline felodipine particles. PXRD results of 10 and 20% loaded patches show no clear evidence of the presence of crystalline drug. The -NH stretching region of the ATR-FTIR spectra of patches also indicates the presence of crystalline felodipine (signature -NH peak at 3667 cm^−1^) in the patches with 30% drug loading and the amorphous (signature -NH peak at 3333 cm^−1^) nature of the drug in the 10 and 20% drug loaded patches, as shown in Fig. 2.

**Fig. 3:**
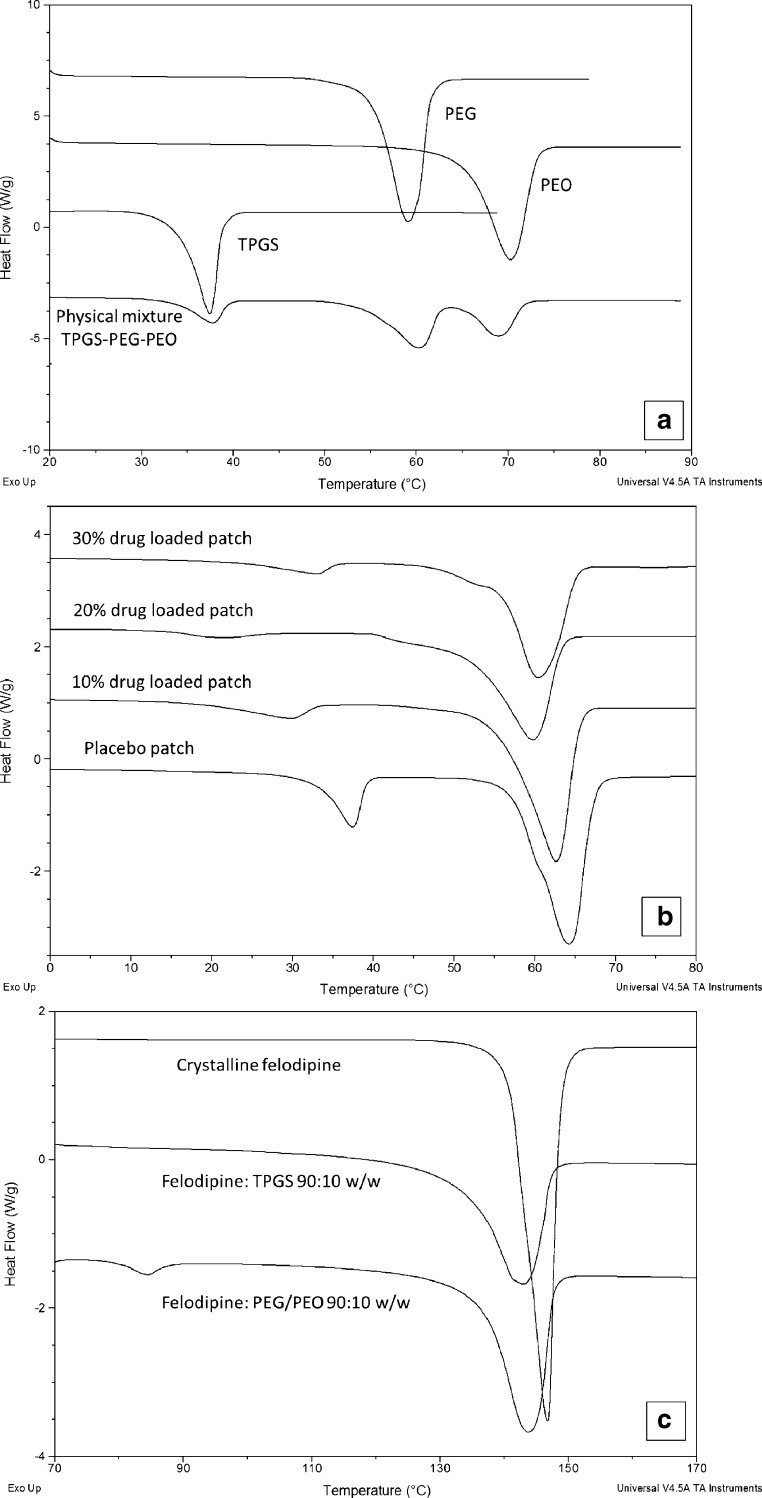
DSC results of (**a**) raw materials and placebo sample; (**b**) felodipine loaded patches with 0–30% *w/w* drug loadings; (**c**) crystalline felodipine in comparison to physical mixtures of 90% crystalline felodipine with 10% TPGS or PEG-PEO.

In order to draw some degree of prediction of phase separation in the dispersions, miscibilities between the excipients and the drug with the excipients were studied using DSC. As shown in Fig. 3a, PEG, PEO and TPGS have melting points at 59.0 ± 0.2, 70.1 ± 0.2, and 37.4 ± 0.4°C, respectively. However after injection moulding, PEG and PEO melting peaks merged into a single peak (Fig. 3b) indicating good miscibility of PEG-PEO and confirming the formation of a single continuous PEG-PEO phase. The DSC thermograms of the physical mixes of the three excipients (with the same ratio as the one used in the placebo patches) retained all of the original melting events of each excipient. This indicates that either the heating rate used is faster than the kinetic process of melting induced mixing between the molten excipients or TPGS has limited miscibility with PEG and PEO. The DSC results of the placebo patches show two melting transitions at 38 and 65°C corresponding to the melting of TPGS and the blend of PEG-PEO, respectively (Fig. 3b). The separate melting of TPGS suggests limited miscibility between TPGS and PEG-PEO. The phase separation of TPGS and PEG-PEO is likely to be the result of the presence of the hydrophobic alpha tocopherol moiety in the TPGS structure.

As a result of the complexity of the composition of TPGS, it is difficult to use theoretical approaches such as calculating the Flory-Huggins interaction parameters using the group contribution method to predict the miscibility of TPGS with the drug and any other excipients used in the patches. An attempt to use the melting point depression method to estimate the miscibility of TPGS and PEG-PEO with the drug also proved unreliable as the dissolution of drug in the molten excipients meant that no melting transition for the drug was observed. Nevertheless the thermal behavior of the physical mixtures of crystalline felodipine and each individual excipient could provide some insight into the miscibility between the drug and the carrier materials. With a low drug:polymer ratio, no melting of crystalline felodipine was detected using DSC due to the melt-dissolution of the drug in the molten carrier material. As seen in Fig. 3c, increasing the ratio of drug:polymer to 90:10 allows the detection of felodipine melting with reduced melting onset and peak temperatures, broader melting peak and reduced melting enthalpy in comparison to pure felodipine (pure crystalline felodipine ΔH_f_ = 76.32 ± 1.44 J/g; crystalline felodipine: (PEG/PEO 4:3) 9:1 ΔH_f_ = 61.46 ± 2.46 J/g; crystalline felodipine: TPGS 9:1 ΔH_f_ = 57.46 ± 0.56 J/g), which indicates a certain degree of miscibility between the drug and each carrier material. The reductions in the onset temperature and enthalpy are more significant for the felodipine:TPGS mixture than the felodipine:PEG-PEO mixture which implies a higher miscibility of the drug with TPGS than PEG-PEO. This leads to the hypothesis that more drug may be solubilized in the TPGS phases than PEG-EPO phase in the processed dispersions.

After felodipine was incorporated in the HME-IM patches, no crystalline felodipine melting was detected by DSC in any patches (data not shown). As earlier, the PXRD and ATR-FTIR results indicated that crystalline felodipine was present in at least in the 30% drug loaded patches, this result suggests that thermal dissolution of crystalline felodipine in the molten excipients occurred during DSC runs. The melting transitions of TPGS and PEG-PEO in the drug loaded patches shifted to lower temperatures than those observed for the placebo patches (Fig. 3b). This melting point depressions of the excipients are likely caused by the dissolved felodipine in the TPGS and PEG-PEO phases during the HME-IM process which may lead to higher level of crystal defects compared to the placebo formulation (34). The melting transition temperatures show drug-loading dependence, as seen in Fig. 3b. It was noted that the lowest melting points of TPGS and PEG-PEO were obtained in the patches with 20% drug loading. This may indicate that the 20% patches contain most dissolved/solubilized drug in the matrices which approaches the saturation or even potentially supersaturation of the drug in the polymer matrices. Further increasing the drug loading to 30% leads to the presence of undissolved/recrystallised crystalline drug accompanied by a shift in the melting peaks of TPGS and PEG-PEO to higher temperatures than were observed in the 10 and 20% loaded patches. However, the melting temperatures of TPGS and PEG-PEO are still lower than those of the placebo suggesting the presence of solubilized drug in the matrices.

Based on the conventional characterization results described above one can conclude that 1) phase separation of TPGS and PEG-PEO is present in all patches; 2) drug loading can affect the phase separation behavior; 3) at a drug loading of 30%, the patches contain phase separated crystalline drug. However, as all systems exhibit phase separation it is important to gain more information on the uniformity and distribution of these separate phases. Here TASC and XμCT are proposed as complementary methods to the more established methods to study the microstructure of the samples.

### TASC Investigation of the Structural Heterogeneity of the Patches

**Fig. 4:**
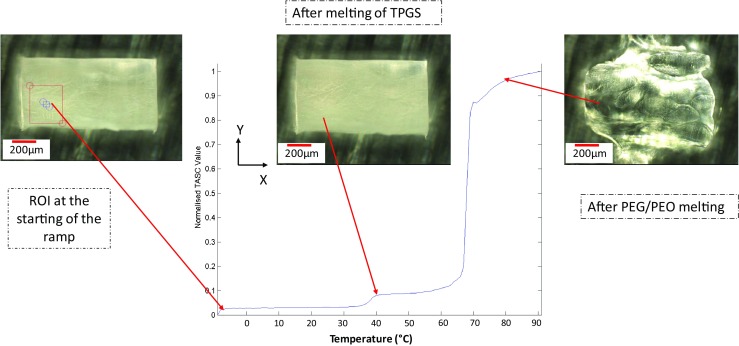
Thermal events of placebo sample detected by TASC at different points (−10°C, 40°C, and 80°C) during the heating ramp at 10°C/ min.

**Fig. 5:**
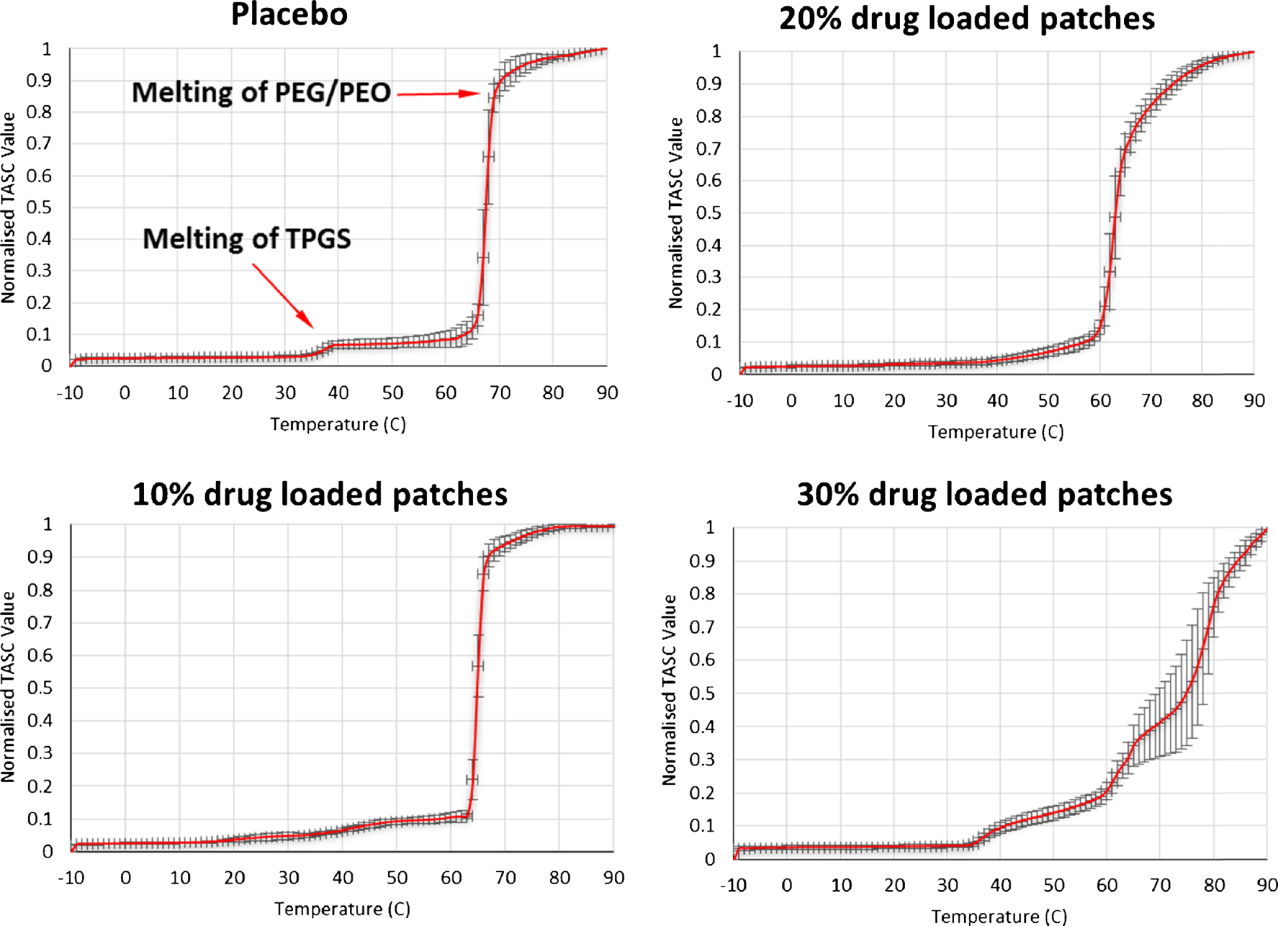
TASC thermograms of the heating cycle of placebo and felodipine loaded patches.

Sequences of images were collected during the heating or cooling of patches using TASC. Initially a region of interest (ROI) was selected (Fig. 4). TASC follows the subtle changes of structure of the selected ROI and converts this information into phase transition signals plotted against temperature. The detailed algorithm of TASC is described elsewhere (20). Figure 5 shows the heating and cooling cycle of the patches measured using TASC. Both the melting of TPGS and PEG-PEO phases can be clearly distinguished on the TASC thermogram of the placebo patches. The transition temperatures are in good agreement with the DSC data. At 10% *w/w* drug loading, the melting of TPGS is less obvious and there is slight lowering in the melting peak of the PEG-PEO phase compared to the placebo sample. For both the placebo and 10% loaded patches, a clear and sharp transition to the plateau of the maximum normalized TASC signal was observed after the melting transition of PEG-PEO. The plateau region is a clear indication of no further changes in the TASC signal of the samples, which can be translated into complete melting in this case. Further increasing the drug loading to 20% *w/w* caused the TPGS melting to almost disappear in the TASC curve and was associated with a further reduction in the melting temperature of PEG-PEO phase. It is noted that after the PEG-PEO melting, the signal approached the plateau region much more gradually in comparison to the TASC results of the placebo and 10% loaded patches.

The TASC results of the patches with 30% *w/w* felodipine content show a complex triple transition. The melting peak of TPGS can be clearly seen at approximately 33°C which is in agreement with the DSC data. Two further melting transitions were detected at 60 and 76°C followed by the absence of the plateau region seen in the placebo and 10% loaded samples. DSC data of the 30% loaded patches only showed the melting of the PEG-PEO phase at 60°C (Fig. 3c). However it is known from the other characterization methods that there were crystalline drug particles present in the 30% patches. Therefore the 76°C transition detected by TASC is likely to be associated with the thermal dissolution of the remaining crystalline drug into the molten matrix. The absence of a plateau region indicates the continuous changes captured by TASC were not completed at 90°C. The poorer reproducibility of data in the high temperature region was also noted in comparison to the results of the samples with lower drug loadings.

**Fig. 6:**
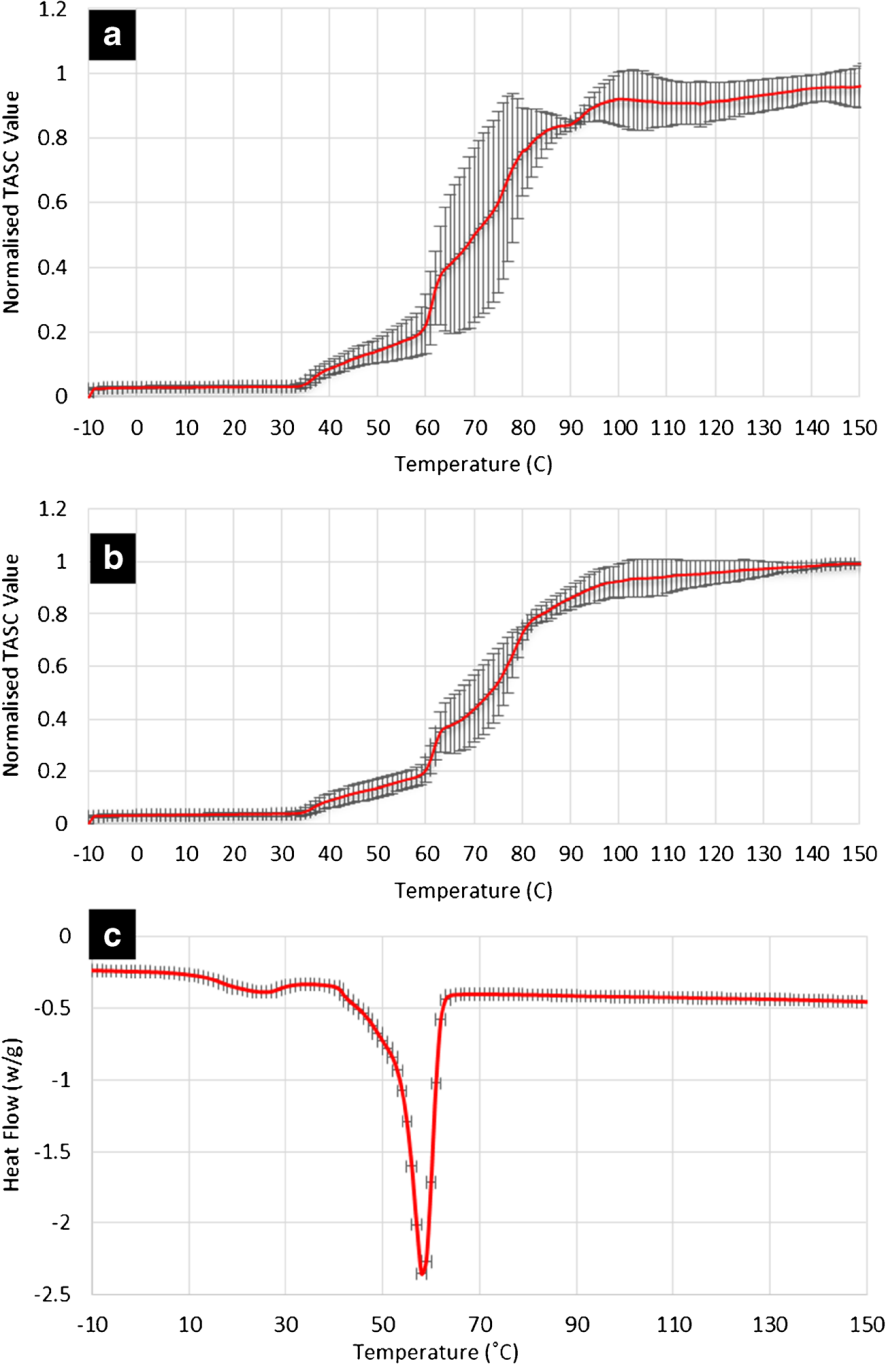
Comparison of the TASC results of the heating cycle of 30% *w/w* felodipine patches using (**a**) small sampling spots (ROIs, approximately between 2.5 × 10^−3^ and 10× 10^−3^ mm^2^), (**b**) larger sampling spots (ROIs, approximately between 40× 10^−3^ and 90× 10^−3^ mm^2^); (**c**) standard DSC averaged thermograms (*n* = 3) for the 30% *w/w* drug loaded samples.

The low reproducibility and failure to reach a plateau with the individual replicates of the 30% *w/w* loaded formulation were further investigated by altering the size of ROI and increasing the terminal temperature of the analysis to above the melting point of crystalline felodipine. As seen in Fig. 6, the reproducibility of the data collected by analyzing small areas (ROIs, approximately between 2.5 × 10^−3^ and 10 × 10^−3^ mm^2^) is lower than that obtained from larger areas (between 40 × 10^−3^ and 90 × 10^−3^ mm^2^). The results obtained using larger tested areas often overlooks the differences present locally on a micro scale (heterogeneity). This is demonstrated by the highly reproducible DSC data in which the samples were tested as a bulk material with no localized information being obtainable (Fig. 6c). The poor reproducibility of the TASC results obtained from small ROIs indicates a high variability in the thermal transitions detected locally. The size of drug crystals detected by SEM is approximately 10–20 μm in diameter which is smaller than the smallest ROI used in this analyses. The thermal properties detected for each ROI is the average of all materials within the area which should therefore be a mixture of drug crystals, excipients and amorphous dispersions of drug dissolved in the excipients. The variation of the thermal properties is likely to represent differing amounts of drug crystals, excipients and amorphous drug dispersions being present in each ROI. This was not observed in placebo and samples with 10% drug loading (Supplementary Information Figure S3). This is a clear indication of the high heterogeneity of the distribution of the separate phases in the patches with 30% drug loading at the micron scale. The attempt of validating such finding by XμCT is described in the next section.

**Fig. 7:**
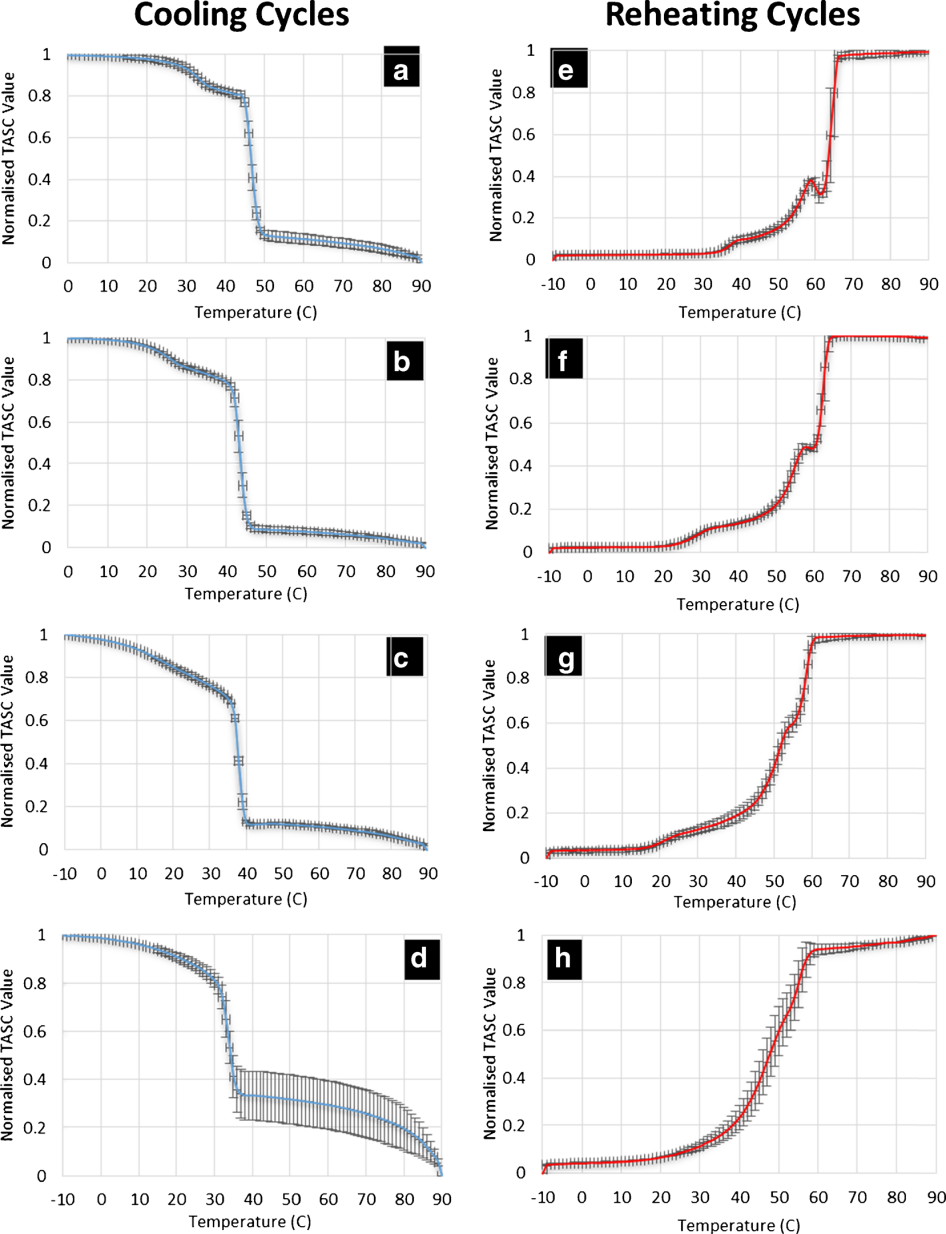
(**a**)–(**d**): TASC thermograms of the cooling cycles of the patches with 0, 10, 20, 30% *w/w* drug loading after first heating; (**e**)–(**h**) TASC thermograms of the reheating cycles of the patches with 0–30% drug loading immediately after the cooling cycles.

**Fig. 8:**
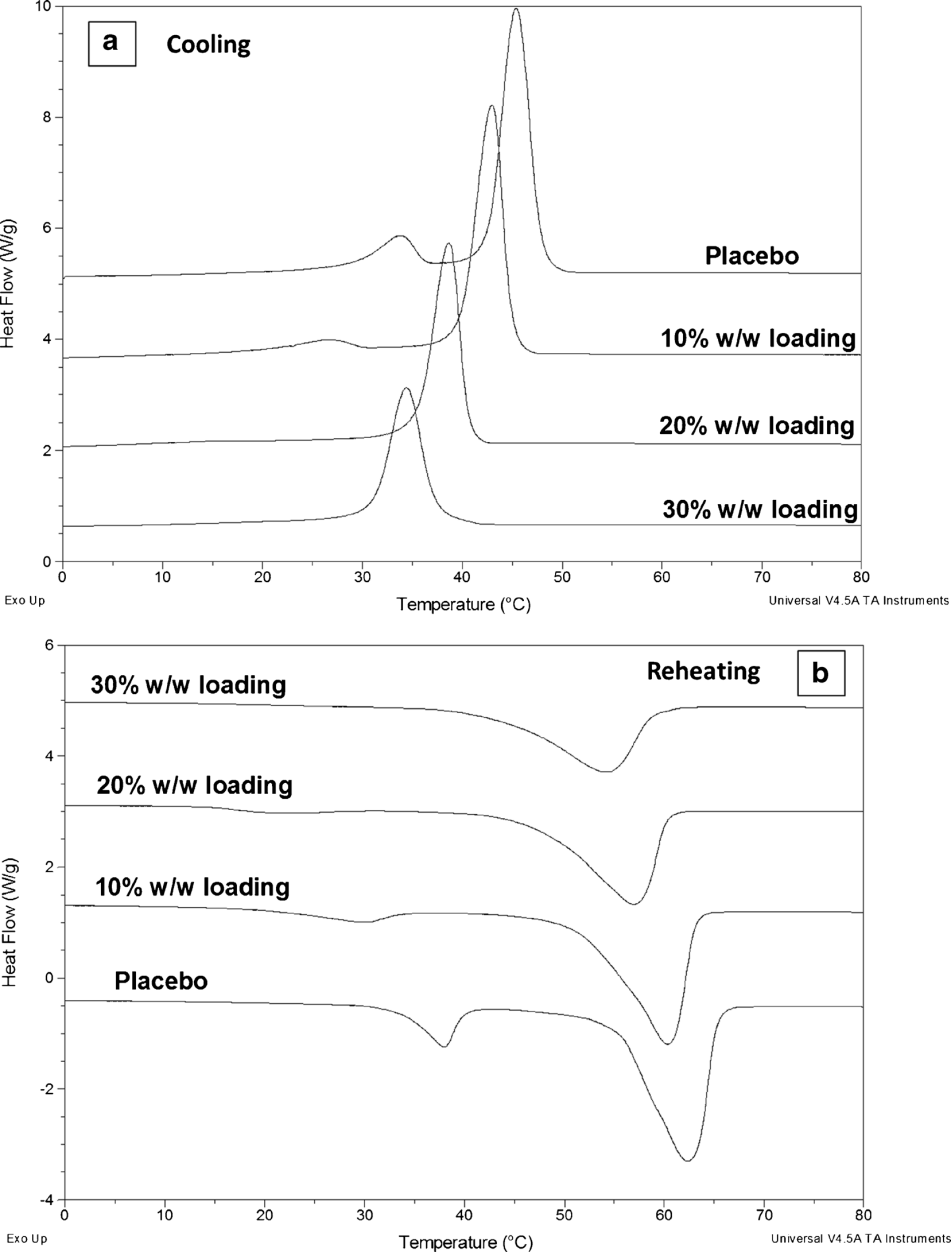
DSC results of the patches with 0–30% drug loading during (**a**) cooling and (**b**) reheating cycles.

To confirm that the slower approach to the plateau of TASC signal for the 30% loaded patches is related to the dissolution of phase separated crystalline drug, the heating was extended to above the melting point of crystalline felodipine. As seen in Fig. 6, a plateau was gradually approached between 100 and 140°C suggesting the slow and temperature dependent thermal dissolution of crystalline felodipine into the molten matrices of TPGS and PEG-PEO. To further investigate the temperature and time dependency of the phase separation behavior of these patches, cooling and reheating cycles of the heated samples were analyzed. As plotted in Fig. 7, double transitions were detected in the cooling cycles of 0–20% loaded patches and the transition temperatures decreased with increasing the drug loading. This agrees well with the corresponding DSC data of the cooling cycle from 160 to 0°C (Fig. 8a), indicating the crystallization of PEG-PEO and TPGS phases. The reduction of the crystallization temperature can be explained by the incorporation of drug in both phases (although not necessarily in equal proportions) which disrupted the crystallization of the excipients. However only a single phase can be seen in the TASC and DSC results of the cooling cycle of the patches with 30% drug loading. This may indicate that TPGS did not crystallise due to the presence of the dissolved drug in these patches.

The TASC results of the reheating cycles show improved reproducibility and new thermal features compared to the heating cycle. As seen in Fig. 7, a new transition at 56–59°C was detected in the patches with 0–20% drug loading. This thermal transition was not detected by DSC at 10°C/min in this study (Fig. 8b). In the literature, it has been reported that using a slower scanning rate would allow the observation of melting of the folded form of PEG 4000 in the presence of drug molecules (35). The fact that it was absent in the measurements of the heating cycle suggests that this transition is highly time dependent. Although the heating cycle was performed on samples freshly prepared by HME-IM, the samples were cooled for at least 1 h prior the measurements being taken. Within this period, the unfolding of PEG chains was already completed and therefore this transition was not detected in the heating cycle. It was also noted that for the samples with 0–20% drug loading, a clear plateau was reached after the sharper melting transitions of the PEG-PEO phases than with the melting transition observed in the heating cycle (Fig. 5). This may be attributed to the high homogeneity and complete lack of a crystalline drug phase separation in these reheated samples. In contrast to the clear identification of TPGS and PEG-EPO melting in the heating cycle, the TASC reheating cycle for the 30% drug loaded samples showed a gradual transition at 50°C and a sharp transition of the signal towards the plateau region. This may indicate that the phase separation of TPGS and PEG-PEO is not completed within the timeframe of cooling-reheating cycle, suggesting that for the 30% drug loaded patches, the kinetics of the phase separation process is slower than the one for the patches with other drug loadings. All transition temperatures observed in the TASC results are in good agreement with the transitions detected by DSC.

### XμCT Analysis of the Internal Microstructure and Spatial Distribution of Crystalline Drug

**Fig. 9:**
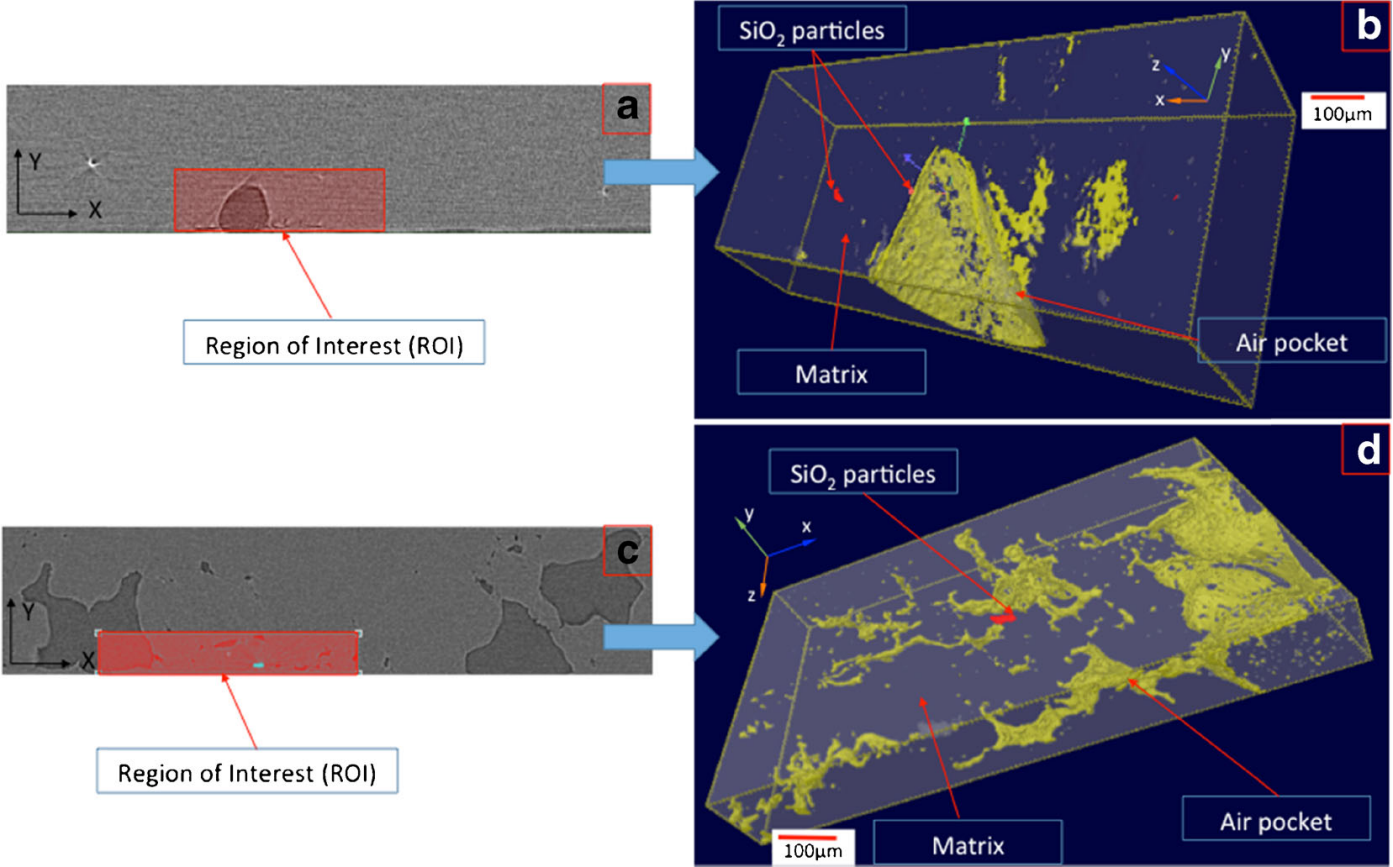
(**a**) XμCT reconstruction of binary image of 10% loaded sample with selected Region of Interest (ROI) for analysis; (**b**) 3D object representing the different components according to their densities present in the selected ROI; (**c**) XμCT reconstruction of binary image of 20% loaded patches with selected Region of Interest (ROI) for analysis; (**d**) 3D object representing the different components according to their densities present in the selected ROI.

**Fig. 10:**
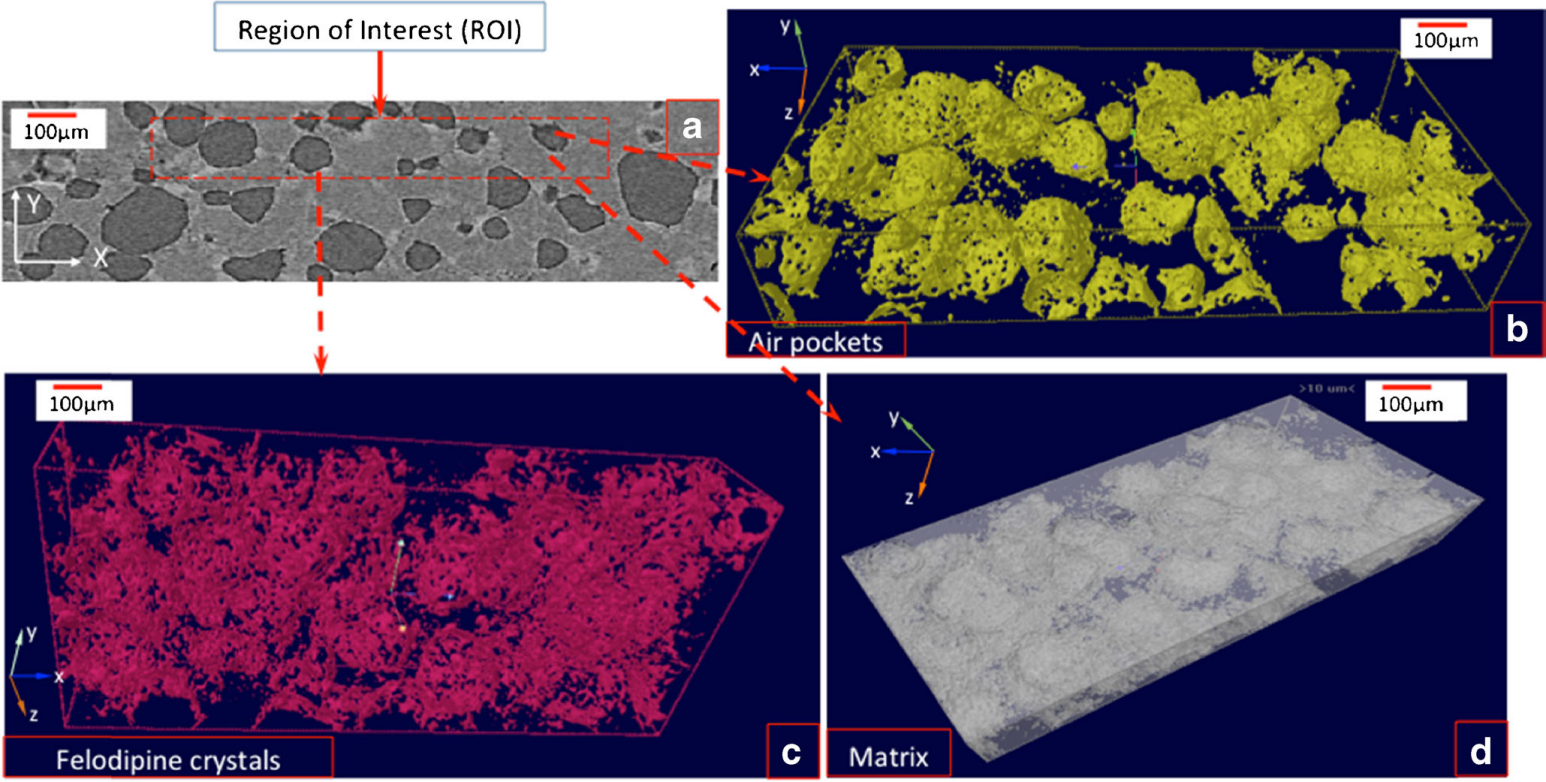
XμCT analysis of 30% loaded sample; (**a**) an example of reconstructed binary image with selected Region of Interest (ROI) for analysis (**b**) 3D object representing the air pockets entrapped in the sample; (**c**) 3D object representing the phase separated crystalline felodipine particles; (**d**) 3D object representing the matrix.

XμCT analysis of the placebo patches revealed that they are pore-free with little interior microstructure at the resolution used in XμCT (Supplementary Information Figure S4). At 10 and 20% drug loading, some internal air pockets are evident as seen in Fig. 9. These occasional air pockets have no defined structure. With increasing the drug loading, the volume fraction of the patches occupied by the air voids was also increased. The few particles with high density shown as bright spots in the matrix were identified as silicone dioxide (SiO_2_) (with a density of 2.65 g/cm^3^), which is an inorganic material present in the powder of PEO at a concentration of 0.8–3% *w/w* as a powder flowability enhancer (36). No other phase separation can be observed in these patches with 10 and 20% drug loading. Although DSC and TASC confirmed the presence of separate TPGS and PEG-PEO phases, both are organic materials with similar elemental composition in their structure which provide no electron density contrast that can be used in XμCT to resolve the different phases. Felodipine has chlorine atoms in its structure which have higher electron density compared to the elements in the excipients. When felodipine dissolved in the excipients as a molecular dispersion, the overall electron density of the local area will be elevated by the presence of felodipine. The fact that no isolated drug clusters can be identified using XμCT for these two patches indicates that felodipine is relatively evenly distributed across the patches. It should also be mentioned that the spatial resolution of XμCT used in this study is within the micrometre range. Therefore, if any drug clusters occur with sizes smaller than few microns, they would not be detectable by XμCT. As seen in Fig. 10, the XμCT images of the patches with 30% drug loading show the presence of clear drug clusters and air voids with well-defined spherical shape. As PXRD and ATR-FTIR spectroscopy results indicated the presence of crystalline drug, it can be stated with some confidence that these drug clusters, represent the crystalline drug particles and be described as crystalline drug particles in the following discussions. The crystalline drug particles are 10–20 μm in diameter, which is similar to the crystals observed using SEM. As seen in Fig. 10a, the crystals (light spots) are more frequently distributed at the interfaces between the air voids and the matrix. This is an interesting feature which was not detected by any other characterisation method used in this study. The DSC results indicate that felodipine has a higher miscibility with TPGS than PEG-PEO and hence drug crystallisation after reaching supersaturation is more likely to occur in PEG-PEO-rich domains than in TPGS-rich domains. Therefore it is reasonable to speculate that these crystalline felodipine-rich areas around the air pockets are also PEG-PEO rich regions.

### XμCT Analysis as a Potential Semi-Quantitative Method to Study Crystalline Drug Content and Heterogeneity

**Fig. 11:**
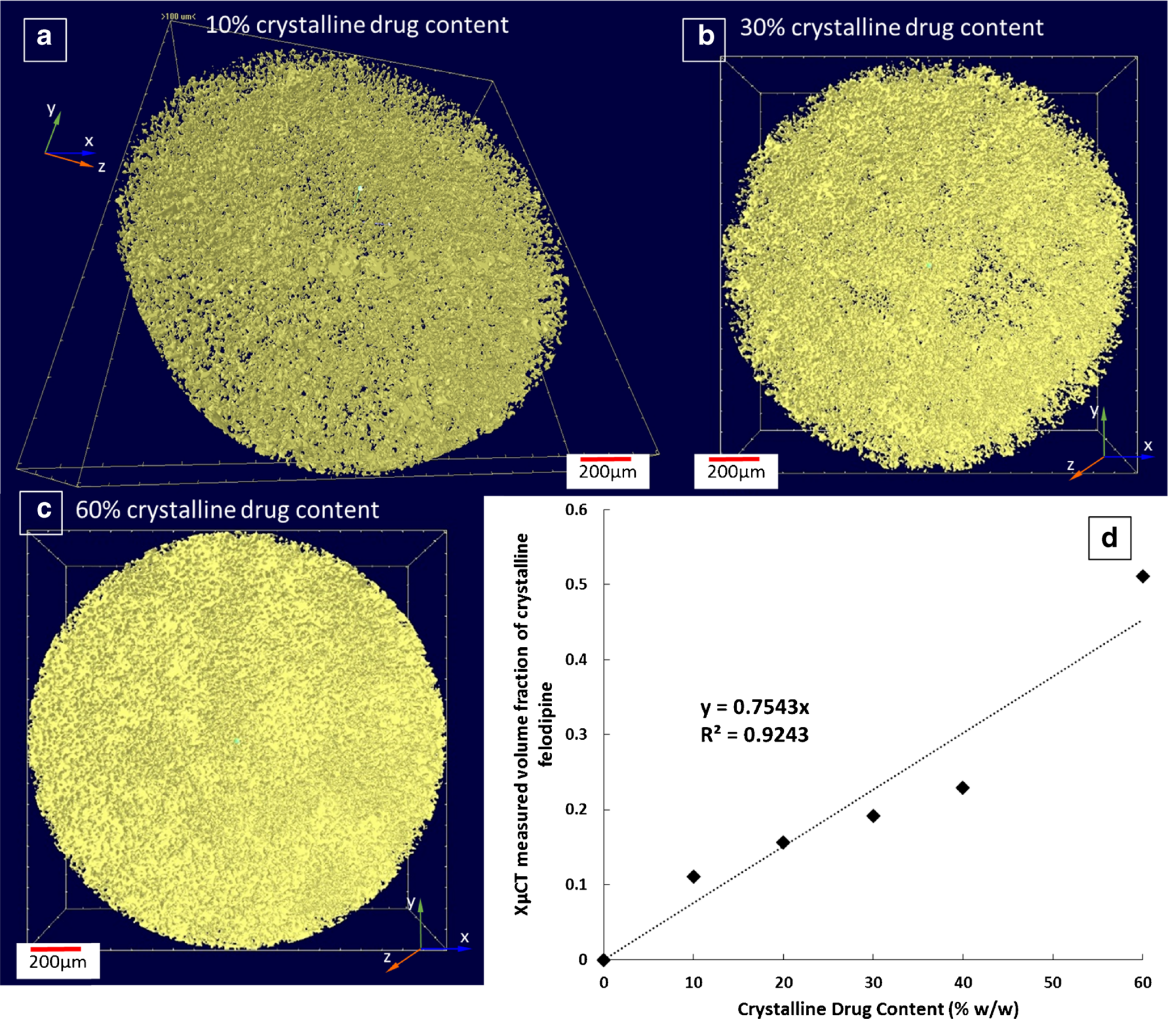
Representative 3D XμCT images of the distribution of crystalline felodipine in the compacts made of the physical mixes of crystalline felodipine-TPGS-PEG-PEO with (**a**) 10%; (**b**) 30%; and (**c**) 60% crystalline felodipine loadings. (**d**) the correlation between crystalline drug content in these compacts and measured volume fraction of felodipine in their 3D XμCT images, which was used as calibration curve for the quantitative estimation of crystalline felodipine in HME-IM patches with 30% drug loading.

In order to further explore the possibility of using XμCT as a quantitative method for characterising phase separation in solid dispersions, compressed compacts of the physical mixes of crystalline felodipine with known drug content (the same drug content as was used in the patches) were prepared and analysed. It should be highlighted that although crystalline drug was used to prepare the physical mixtures, only differences in the chemical makeup of the drug and excipients can be observed by XμCT not their physical form. As seen in Fig. 11, crystalline felodipine particles are evenly distributed across the matrices. The volume fraction of the space occupied by the crystalline drug particles can be measured and the values for the compacts with 10–60% crystalline drug loading were plotted against the known drug content (Fig. 11d). It was noted that the linearity of the correlation was not ideal (with a regression R^2^ of 0.92). Therefore these results should be regarded as semi-quantitative. It was noted that the compacts were much softer after compression than normal solid tablets and the surfaces of the compacts were slightly tacky. This softening indicates the lowered melting point of the mixture which could be caused by solubilisation of crystalline drug in the low melting excipients such as TPGS during the high-pressure compression process. This may explain why the 60% drug loaded physical mixture shows more deviation from the linear correlation in comparison to results obtained from the 10–40% drug loaded physical mixtures. Using systems that do not have dissolution or physical form changes of the drug during compaction with excipients may improve the accuracy and linear correlation between drug loading and XμCT measured volume. Nevertheless the attempt of using the correlation as a calibration curve was made to estimate the amount crystalline drug in the HME-IM patches with 30% drug loading. The volume fraction of the crystalline drug particles observed in Fig. 10 is 0.078. Using the linear correlation shown in Fig. 11d the weight fraction of crystalline drug can be calculated as 10.3% (*w/w*). This indicates that 19.7% felodipine was molecularly dispersed in the matrices in the HME-IM patches with 30% drug loading. As no crystalline drug was detected in the HME-IM patches with 20% drug loading, it indicates that the 20% is close to the saturation of the solubility of felodipine in the matrices. Therefore for the patches with 30% loading, approximately 10% drug should be phase separated as crystalline drug. The XμCT quantitative estimation agrees well with this.

**Fig. 12:**
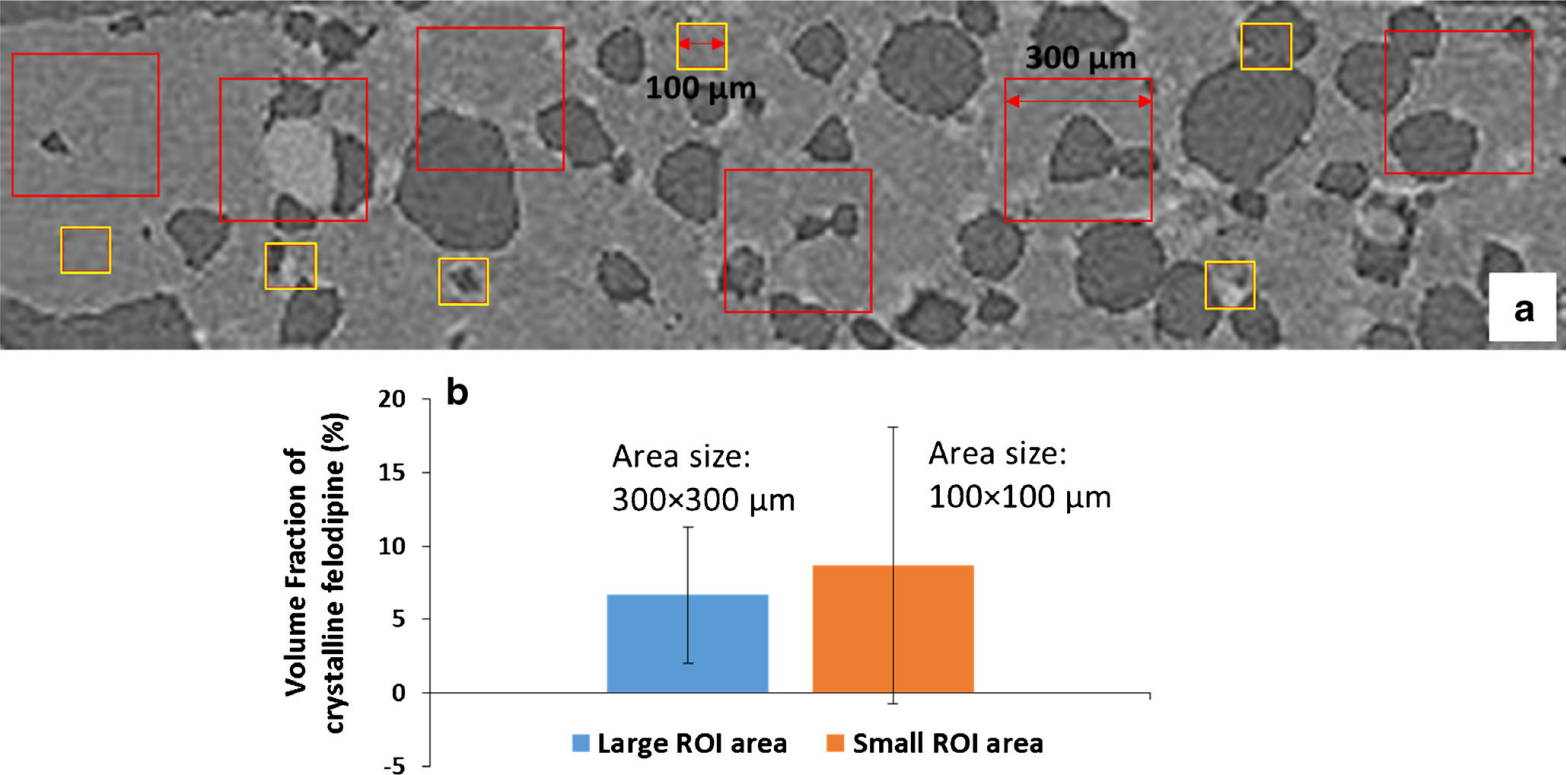
Estimation of heterogeneity by XμCT: (**a**) Illustration of the selection of a range of ROIs with different sizes on a representative 2D XμCT image of the patches with 30% drug loading; (**b**) the comparison of the calculated volume fraction of crystalline felodipine in the ROIs with large (300 × 300 μm) and small (100 × 100 μm) areas.

The heterogeneity of the patches with 30% drug loading was studied using XμCT in order to make comparison with the measurements on heterogeneity by TASC. The same methodology used with TASC for measuring heterogeneity was adopted and areas of interests (ROI) with various sizes were taken from 2D XμCT images. Using the quantitative calibration described above, the amount of crystalline felodipine in each ROI were calculated. As shown in Fig. 12a, a single XμCT slice (grey scale image) was used and 6 small (100 × 100 μm equivalent to 10 × 10^−3^ mm^2^) ROIs and 6 large ROIs (300 × 300 μm equivalent to 90 × 10^−3^ mm^2^) were randomly selected and analyzed. These areas are similar in size to the ones used in TASC measurements. Same thresholding procedure was adapted for the estimation of the volume fraction of phase separated crystalline felodipine in all of these ROIs. It can be seen in Fig. 12b that the amounts of crystalline felodipine measured in larger ROIs have lower standard deviation in comparison to those measured in the smaller ROIs indicating the high heterogeneity at the scale of 100 × 100 μm. This finding agrees well with the results obtained by TASC and confirm that integrating large areas reduces the sensitivity to heterogeneity and explained why heterogeneity is not detected by DSC analysis.

### *In Vitro* Drug Release from the HME-IM Patches

**Fig. 13:**
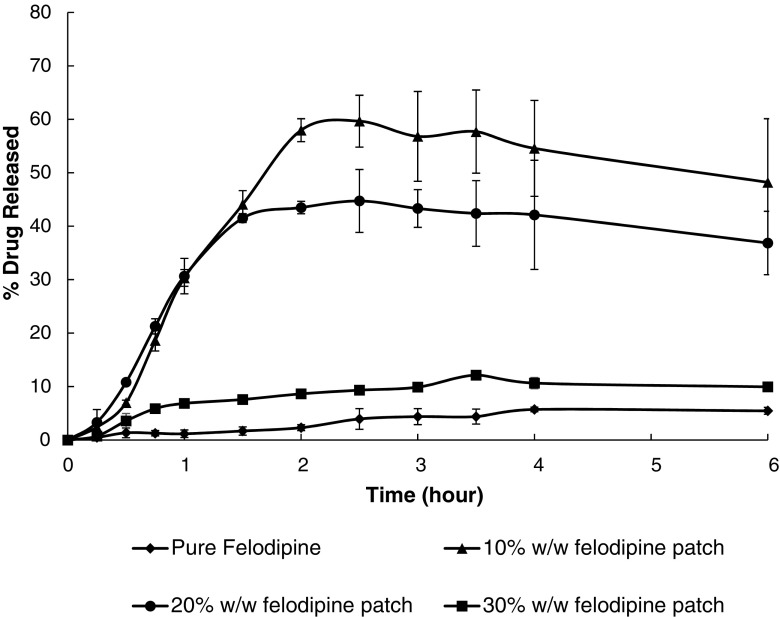
Unidirectional release profile of felodipine from buccal adhesive patches performed under non-sink conditions in pH 6.8 phosphate buffer saline.

*In vitro* unidirectional drug release data of the patches with different drug loading tested under non-sink conditions are shown in Fig. 13. For the 10 and 20% *w/w* patches, 10–15 fold increases in maximum drug release were achieved within 2–2.25 h in comparison to the crystalline drug alone. This may be attributed to fact that the majority of drug in these two formulations is in the amorphous state, which led to faster dissolution. However, with increasing the drug loading to 30%, the increase in drug release reduced to only 2-fold in comparison to the crystalline drug. The presence of phase separated crystalline drug located in at the interface of the air pockets (likely to be the PEG-PEO domains) in the patches is likely to be responsible for this result. The dissolution results indicate that the phase-separated carrier systems that contain no crystalline drug can significantly improve drug release. Even with only one side of the intact patches in contact with the dissolution media, this dissolution enhancement is comparable with other binary solid dispersion systems reported in the literature where milled extrusion powders with much higher total surface area for dissolution were used (9).

## Conclusion

This study introduces the use of two novel characterisation methods for studying phase separation behaviour in pharmaceutical solid dispersions, TASC and XμCT. The characterisation techniques were challenged by a set of complex multi-component solid dispersions containing TPGS, PEG, PEO and the model drug felodipine. The results confirmed that both techniques not only could provide complementary information to conventional characterisation tools, such as DSC, PXRD, ATR-FTIR and SEM-EDS to reveal the correlation between drug-excipient miscibility and phase separation, but also are able to provide a new and important understanding of the heterogeneity and distribution of separate phases in the systems. TASC allowed rapid identification of heterogeneity in the dispersions containing phase separation but does not have the capability of analysing the spatial distribution of the phases. As a non-destructive technique, XμCT analysis provided the 3D microstructure of the interior of the patches and the spatial distribution of the separated phases. The drug release results reflected the negative impact that phase separation of drug clusters had on the dissolution of the poorly soluble model drug. This detailed understanding of the dispersions will provide confidence in product quality of dispersions formulations. However, it should be highlighted that XμCT cannot be used as identification method on its own for distinguishing crystalline and amorphous drug domains. The first attempt of using XμCT as a quantitative method to estimate phase separated drug clusters (identified as crystalline drug with confirmation by PXRD and ATR-FTIR) in processed formulations indicated its potential application for such purposes. However the results reported here can only be regarded as semi-quantitative. Further studies are needed to validate XμCT as a quantitative method.

## Electronic supplementary material

Below is the link to the electronic supplementary material.

ESM 1 (DOCX 1453 kb)

## Abbreviations

3D: Three dimensional
AFM: Atomic force microscopy
ANOVA: Analysis of variance
ATR-FTIR: Attenuated total reflection fourier transform infrared spectroscopy
DSC: Differential scanning calorimetry
EDS: Element dispersive spectroscopy
HME-IM: Hot melt extrusion-injection moulding
IM: Injection moulding
IR: Infrared
MTDSC: Modulated temperature differential scanning calorimetry
PEG: Polyethylene glycol
PEO: Polyethylene oxide
PXRD: Powder x-ray diffraction
ROI: Region of interest
SEM: Scanning electron microscopy
SEM-EDS: Scanning electron microscopy- element dispersive spectroscopy
TA: Thermal analysis
TASC: Thermal analysis by structural characterization
TPGS: Tocopheryl polyethylene glycol succinate
TTM: Transition temperature microscopy
USP: United States pharmacopeia
UV–VIS: Ultraviolet–visible
XμCT: X-ray micro computed tomography
