# Supplementary material for: Characterization of Heterogeneity and Spatial Distribution of Phases in Complex Solid Dispersions by Thermal Analysis by Structural Characterization and X-ray Micro Computed Tomography
Source: Pharm Res. 2016 Apr 19;34(5):971–89. doi: 10.1007/s11095-016-1923-3 (PMC5382188; doi:10.1007/s11095-016-1923-3)

**Supplementary Information**

**Characterization of heterogeneity and spatial distribution of phases in complex solid dispersions by thermal analysis by structural characterization and X-ray micro computed tomography**

Muqdad Alhijjaj^1,2^, Samy Yassin^3^, Axel Zeitler^3^, Nikolaus Wellner^4^, Mike Reading^5^, Peter Belton^6^, and Sheng Qi^1*^

1. *School of Pharmacy, University of East Anglia, Norwich, Norfolk, UK, NR4 7TJ*
2. *College of Pharmacy, University of Basrah, Basrah, Iraq*
3. *Department of Chemical Engineering and Biotechnology, University of Cambridge, Pembroke Street, Cambridge CB2 3RA, UK*
4. *Institute of Food Research, Norwich Research Park, Colney Lane, Norwich, Norfolk, NR4 7UA*
5. *Department of Chemical Sciences, University of Huddersfield, Queensgate, Huddersfield, UK, HD1 3DH*
6. *School of Chemistry, University of East Anglia, Norwich, Norfolk, UK, NR4 7TJ*

Correspondence: Sheng Qi, [sheng.qi@uea.ac.uk](mailto:sheng.qi@uea.ac.uk); Fax number: +44 1603592023

**Dissolution method development and validation:**

As this study represents the design of drug delivery system for felodipine through the buccal mucosa and because the product is currently in the early stages of development, the following validation procedures were used to optimize the *in vitro* release study:

1) Filter medium suitability:

Filtration using the filter unit (0.45 um pore size) was compared with centrifugation using (4500 rpm for 10 min) to investigate the suitability of the filter units used in this study. The filtered and centrifuged samples withdrawn during the same time interval of peak dissolution concentrations were used to exclude the probability of filter medium unsuitability. The results indicated that there is no significant difference in the concentration detected by UV-Vis spectrophotometry at 363 nm. These results validates the use of the filter unit used in this study and proved that there is no adsorption of the drug in the filter. Furthermore, during the course of the experiment the researcher adapted a method of not using the first volume withdrawn as recommended by the recommended references [1].

2) Solubility and stability of the drug in the dissolution medium:

The saturated solubility of crystalline felodipine in the dissolution medium used in this study was determined in our previous work [2]. In this study, we stated that the experiment was done using non-sink condition. We use this approach as our previous work showed clearly that using surfactant with the dissolution medium (under sink condition) can greatly affect the release profile of poorly water soluble drug felodipine. Thus, we decided to use the non-sink condition because we believe that the release profile obtained is more reflecting the actual state compared to the use of surfactants to achieve sink conditions. On the other hand, the results obtained from this study under non-sink showed an improvement of the dissolution properties of the drug which is one of important key finding stated in this study.

3) Choosing the medium and the volume:

There is no standard method to perform the release profile for this kind of preparation in the pharmacopeia. The used parameters were adapted from the peer reviewed articles most suitable for serving the purpose of this study. As an example of this, we used buffer medium (phosphate buffer saline PBS) with pH of 6.8 to represent the buccal environment. There is also no specified volume for the dissolution medium stated in the pharmacopeia as well.

4) Choosing an Apparatus:

Paddle over disc method was used to evaluate this kind of drug delivery system. In this method, a unidirectional drug release profile can be achieved to simulate the situation at the site of absorption.

**Reference:**

[1] USP36-NF31, Chapter <1092> The Dissolution Procedure: Development and Validation. United States Pharmacopoeia 36/National Formulary 31, Baltimore, MD, USA; 2014: 735.

[2] Alhijjaj M, Bouman J, Wellner N, Belton P, Qi S. Creating Drug Solubilization Compartments via Phase Separation in Multicomponent Buccal Patches Prepared by Direct Hot Melt Extrusion–Injection Molding. Mol. Pharm. 2015;12(12):4349-62.

**Figure S1.** Elemental analysis of drug loaded patches


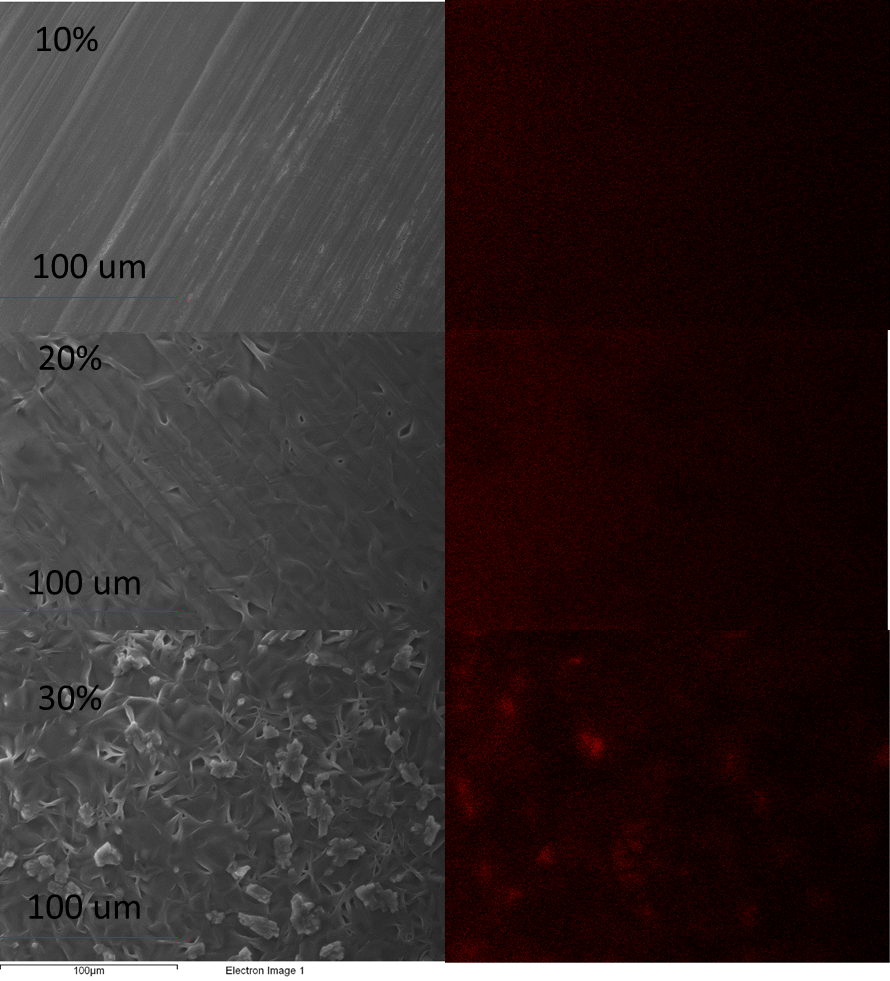


**Figure S2.** PXRD patterns of excipients, placebo patches and HME-IM buccal patches with 10-30% (w/w) felodipine loadings. The dashed line boxes highlight the regions where changes in diffractions peaks associated with the drug and the polymers are observed.


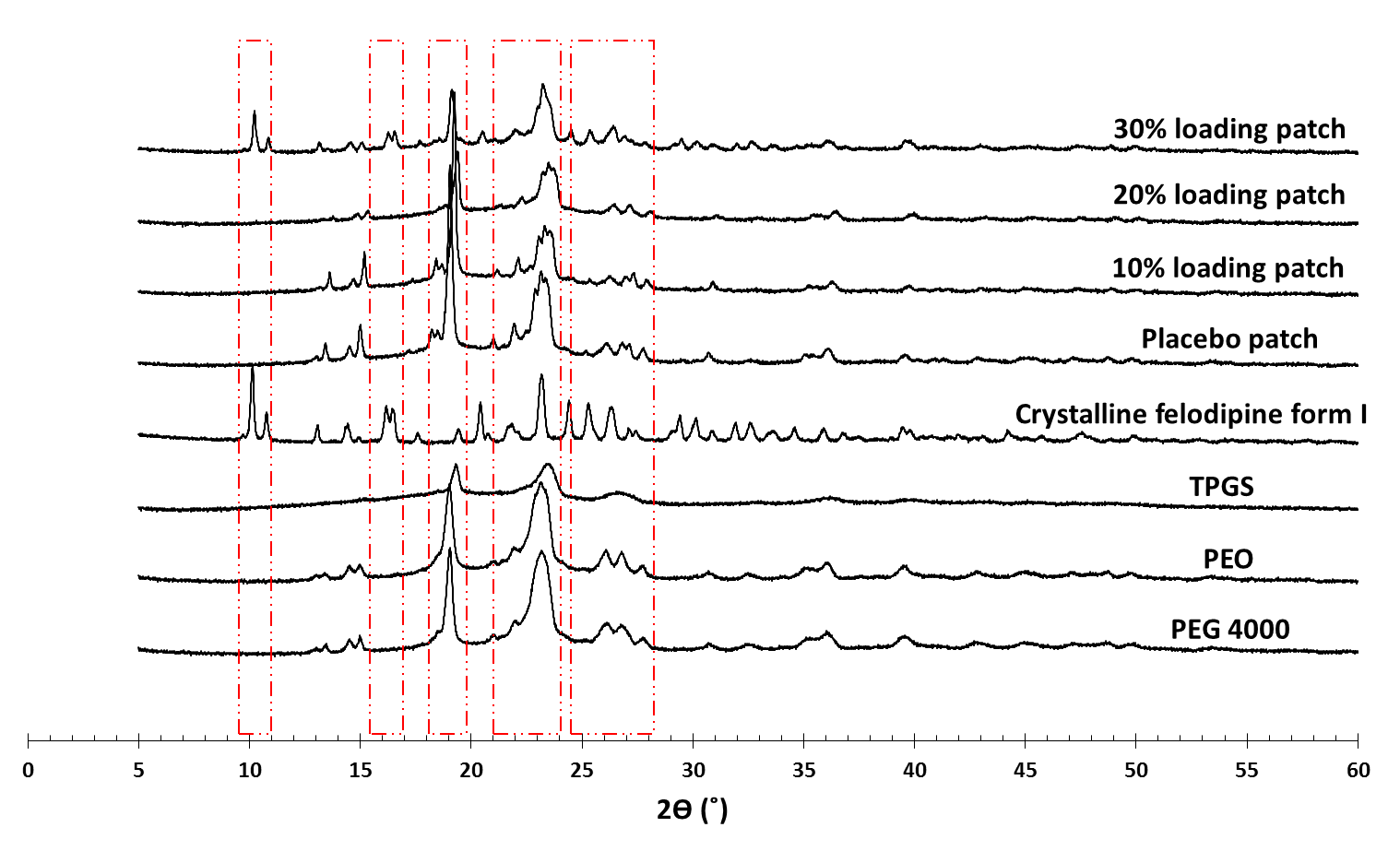


**Figure S3.** The TASC results of the heating cycle of 10% w/w felodipine patches using a small sampling area (top panel) and a larger sampling area (bottom panel). It can be seen that there is no obvious difference in the error bars of the data collected from the two areas with different sizes indicating the high homogeneity of the samples with low drug loading.


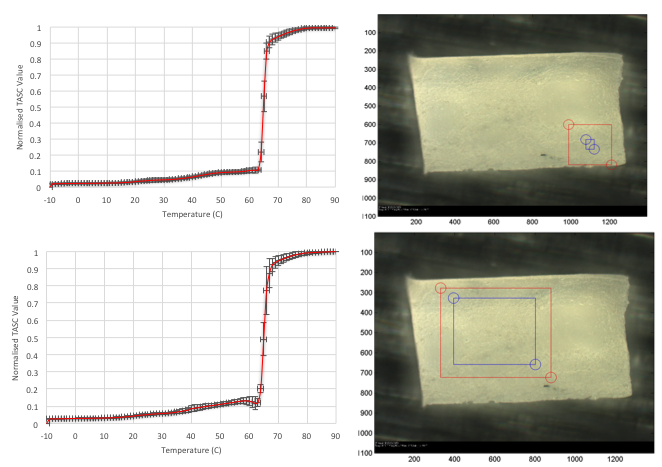


**Figure S4.** Micro-CT analysis of placebo sample; A) an example of reconstructed binary image; B) selected Region of Interest (ROI) for analysis C) 3D object representing the different components according to their densities present in the selected ROI.


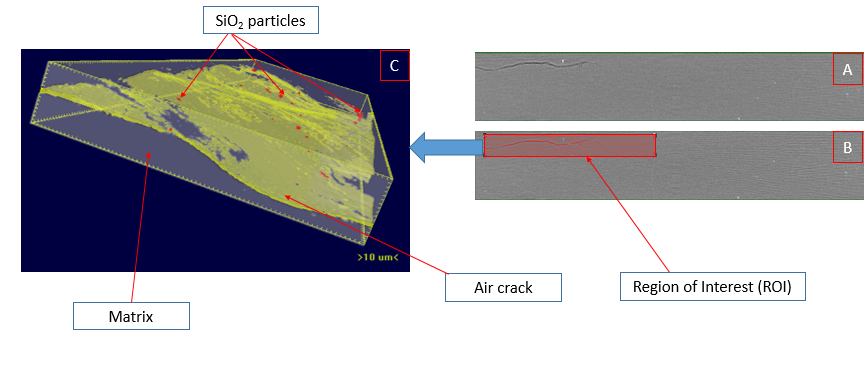

Supplement: Supplementary file 1 — (DOCX 1453 kb) [file 11095_2016_1923_MOESM1_ESM.docx]
